# High-Performance Materials Improve the Early Shrinkage, Early Cracking, Strength, Impermeability, and Microstructure of Manufactured Sand Concrete

**DOI:** 10.3390/ma17102465

**Published:** 2024-05-20

**Authors:** Mingming Zhang, Shan Gao, Tong Liu, Shuyu Guo, Shuotian Zhang

**Affiliations:** 1 School of Civil Engineering, Xijing University, Xi’an 710123, China; mingzai86782021@163.com (M.Z.); lt15214083131@126.com (S.G.); mmzhang_xian@163.com (S.Z.); 2 School of Civil Engineering, Xi’an University of Architecture and Technology, Xi’an 710055, China; 3 Key Laboratory of Structures Dynamic Behavior and Control of the Ministry of Education, Harbin Institute of Technology, Harbin 150090, China

**Keywords:** manufactured sand concrete, shrinkage reduction, crack resistance, inhibition mechanism, microstructure

## Abstract

The poor early shrinkage and cracking performances of manufactured sand concrete, waste powder concrete, and recycled aggregate concrete are the main difficulties in engineering applications. To solve these problems, early shrinkage and cracking, strength, and impermeability tests were performed on high-volume stone powder manufactured sand concrete mixed with fly ash and slag powder (FS), a shrinkage-reducing agent (SRA), polyvinyl alcohol (PVA) fibers, and a superabsorbent polymer (SAP). Furthermore, the microstructures and pore structures of these concretes were revealed using nuclear magnetic resonance (NMR) and scanning electron microscopy (SEM). The results showed that the mixture of FS, SRA, PVA fibers, and SAP could effectively inhibit the shrinkage strain and cracking area of the concrete. The effect of the SAP on reducing the early shrinkage of the concrete is the greatest, and the shrinkage strain can be reduced by 76.49%. The PVA fibers had the most obvious effect on inhibiting the early cracking of the concrete, and the total cracking area was reduced by 66.91%. Significantly, the incorporation of the FS can improve the particle gradation and the pore structure and improve its compactness. The PVA fibers not only provide good carriers for cement-based materials but also enhance the bonding force between the particles inside the concrete, filling the pores inside the concrete, inhibiting the loss of water, and reducing the generation of internal microcracks. The FS and PVA can reduce the shrinkage and cracking risk and improve the strength and impermeability of the concrete. Although the SRA and SAP can reduce the shrinkage and cracking risks, it will lead to a significant decrease in the later strength and impermeability. The main reason is that the SRA leads to an increase in micropores in the matrix and microcracks near the aggregate, which are not conducive to the development of the strength and penetration resistance of the MS. Similarly, the SAP can promote the rapid formation of ettringite (Aft) at an early age and improve the early shrinkage, early cracking, and early strength of the concrete. However, with an increase in age, the residual pores, after SAP dehydration, will cause the deterioration of the concrete pore structure, resulting in the deterioration of the strength and impermeability.

## 1. Introduction

The market demand for manufactured sand (MS) has increased with the development of infrastructure construction in recent years [1], and MS has gradually become a green economic substitute for river sand in concrete. Generally, a wealth of fine particles is produced during the production process of MS. These fine particles are smaller than 75 μm in the MS and are called stone powder (SP) [2,3]. In recent years, the influences of SP as a fine aggregate or cementitious material substitute on the workability [4,5], mechanical properties [6,7,8,9], volume stability [1,10], and durability [11,12,13,14] of concrete has been extensively studied by researchers. It is commonly believed that a certain amount of SP, as a fine aggregate or cementitious material substitute, can increase the volume of cement slurry in concrete, improve the workability, fill the gaps between coarse aggregates and fine aggregates, and improve the overall density of concrete. However, a high-volume SP leads to a significant reduction in the volume stability and some durability, such as early-age shrinkage, early-age cracking, and resistance to chloride ion penetration [1]. Ahmed and Marar [7] and Celik and El-Kourd [6] believed that the water demand of concrete mixtures increases with SP content, while the adhesion and water retention of concrete increase. Liu et al. [1] reported that an appropriate amount of SP is beneficial for improving the comprehensive performance of the MS concrete, but excessive SP will lead to some performance degradation. Zhang et al. [10] also noted that excessive SP leads to severe deterioration in the performance of the MS concrete, especially early-age shrinkage and cracking resistance. In addition, Soroka and Stern [15] demonstrated the filling effect of SP on cement-based materials in terms of the microstructure for the first time. Since then, the nucleation effect, dilution effect, and chemical effect of SP [16,17,18,19,20] have also been demonstrated. Liu et al. [1] further proposed that excessive SP also has adverse effects on cement-based materials, such as microcrack effects and connected pore effects.

Approximately 15 wt.% of SP is produced during the production of MS. Although the content of SP can be reduced by cleaning or screening, reduced SP content not only requires material consumption and resource waste, but also its subsequent SP landfill disposal can cause severe natural and ecological damage. Therefore, the full utilization of SP in MS is an urgent problem to be solved, and increasing the content of the SP in MS concrete and increasing the application of concrete with the high-volume SP have become hot topics in the concrete industry. At present, improving the early volume stability performance of the MS concrete is the key to promoting the engineering application of high-volume-SP concrete [1,10,21]. Significantly, Golewski [22] reported the effects of CFA additives on the water absorption level of waste cement concrete. The reduction and optimization of water absorption can effectively improve the cracking and shrinkage properties of concrete. Yun et al. [23] evaluated the effects of rheological and physicomechanical properties on the plastic cracking of high-performance shotcrete concrete containing various supplementary cementitious materials. Furthermore, some other studies [24,25,26,27,28,29,30,31,32] also show that in addition to traditional measures for reducing the shrinkage and cracking of concrete from the raw materials, such as coarse aggregates, fine aggregates, and water reducers [24,25], new material methods, such as adding FS [26,27,28] or a high-efficiency SRA [29,30] and fiber toughening [31,32], and internal curing methods, such as SAP [21,25], can also be used to reduce the shrinkage and cracking of concrete. However, there are a few public reports on the inhibitory effect and inhibitory mechanism of the above methods on the early volume stability of the MS concrete with the high-volume SP, and the influences of these methods on the strength and durability of hardened concrete are also unknown.

Considering the above problems, this study takes limestone MS concrete with a content of 20 wt% SP as the research object, and the inhibiting effects on concrete shrinkage and cracking by adding FS, SRA, PVA fibers, and SAP were analyzed. The influences of different shrinkage and crack suppression measures on the early shrinkage, cracking, mechanical properties, and chloride ion penetration resistance of the high-volume-SP concrete were analyzed. Furthermore, SEM tests and NMR were used to test the microstructures and pore structures of different concrete samples, and the effects and mechanisms of different measures on inhibiting shrinkage and cracking in concrete were analyzed. The effects and mechanisms of these four measures on the early shrinkage, cracking performance, mechanical properties, and penetration resistance of the concrete are revealed and analyzed.

## 2. Materials and Methods

### 2.1. Materials

**Figure 1:**
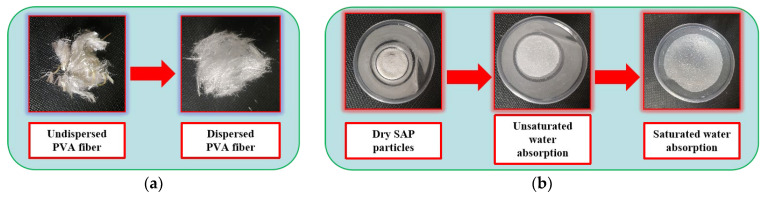
PVA fibers and SAP: (**a**) PVA fibers; (**b**) SAP.

The cement performance indicators of P·O 42.5 and the main properties of limestone MS are shown in Table 1 and Table 2. The limestone MS was used as a fine aggregate; the SP is the fine powder produced by the MS during the crushing process. The coarse aggregate was 5–20 mm graded crushed stone; the shrinkage-reducing and crack-resistant materials used in this test were commercial F-class II fly ash, S95 slag powder, and a commercial polyether shrinkage-reducing agent. Cement, fly ash, slag, SRA, and coarse aggregate were all purchased in the China commercial market. The PVA fibers, having a length of 12 mm, diameter of 40 μm, and density of 1290 kg/m^3^, were purchased from Shanghai Chenqi Chemical Technology Co., Ltd. (Shanghai, China), as shown in Figure 1a. The SAP was purchased from the Yixing Kexinde Chemical Co., Ltd. (Yixing, China); the main component was an acrylamide copolymer, and the particle size was 50−80 mesh (300−180 μm) for the white crystal particles, as shown in Figure 1b. The polycarboxylate superplasticizer, with a water reduction rate of 28%, was obtained from Shanxi Feike New Material Technology Co., Ltd. (Xi’an, China), and the water that was used was ordinary water in the laboratory.

### 2.2. Methods and Mixtures

It is necessary to add SP to the raw material MS to reach a 20% SP content before the test. The SP (fine powder < 75 μm) is produced from MS during the crushing process. The MS with a high-volume SP content of 20% was used to prepare the base group concrete (C0), which was not subjected to any inhibition measures. Furthermore, four different methods for inhibiting concrete shrinkage and cracking were tested in this study. In the first mix (C1), 20% fly ash and 10% slag powder were used to replace the same mass of cement at the same time, which are widely used and recognized in practical engineering at present. The second mix (C2) adopted an SRA content of 1.5 wt.% of the mass of the cement. For the third mix (C3), PVA fibers were used, and the fiber content was 0.2 vol% of the concrete volume. The SAP used in the fourth mix (C4) was 0.2 wt.% of the mass of the cement. The concrete mix ratio of each test group is shown in Table 3.

It is worth noting that the direct incorporation of SAP will have a great impact on the workability of the concrete [1,10]. Therefore, it is necessary to use the SAP pre-absorbent before the incorporation. The pre-absorbent ratio is m(SAP):m(water) = 1:20, and the amount of water inhaled by the SAP should be deducted from the total amount of water used for mixing concrete. Importantly, the five groups of concrete in this test should theoretically maintain the same amount of the water-reducing agent. However, the test process revealed that the mixing water consumption of the C4 concrete, after SAP pre-absorption, decreased, resulting in a significant reduction in the working performance of the C4 concrete. Meanwhile, C1 added with FS and C3 supplemented with PVA fibers also exhibited a decrease in the fluidity of the concrete. Therefore, the concrete slump of each test sample being close to that of the C0 concrete (200 ± 20 mm) was ensured by increasing the superplasticizer so that the comparison of the test results was more credible and reasonable [33,34].

### 2.3. Testing

#### 2.3.1. Shrinkage and Crack Tests

The 3–72 h shrinkage curve and 1-day cracking results of the concrete samples were tested according to the non-contact method and the slab method of the GB/T 50082-2009 standards [35]. The shrinkage test specimen is a prism with a size of 100 mm × 100 mm × 515 mm, and the test results are expressed as the average values of three specimens. The cracking-test specimen is a flat plate with a size of 800 mm × 600 mm × 100 mm, and the test results are expressed as the average values of three specimens. The equipment, steps, and details of the early-age shrinkage and cracking tests can be found in references [1,10,35].

#### 2.3.2. Mechanical Properties and Impermeability

The compressive strength (*f*_cu_) and flexural strength (*f*_f_) of the concrete were tested according to the methods specified in the GB/T 50081-2019 standards [36]. Cubic specimens, for *f*_cu_, of 100 × 100 × 100 mm and prism specimens, for *f*_f_, of 100 mm × 100 mm × 400 mm were prepared. The 28-day electric flux value was calculated for the concrete according to the electric flux method of the GB/T 50082-2009 standards [35]. The electric flux specimen is a cylinder with a height of 50 mm and a diameter of 100 mm. Under the curing conditions (T = 20 ± 2 °C, RH = 95 ± 5%), the samples were measured after curing for 7 or 28 days.

#### 2.3.3. Nuclear Magnetic Resonance (NMR) Tests

A MacroMR12-150H-I analysis system was used in the NMR tests, and the size of the NMR specimen was completely consistent with that of the electric flux specimen. The sample was subjected to a negative-pressure-vacuum water retention treatment after curing and then the sample was wrapped in a plastic film for MacroMR12-150H-I testing. The NMR test steps and methods can be found in reference [10].

#### 2.3.4. Microstructure Test

A sample of approximately 5 mm × 5 mm was intercepted on a test piece after the compressive strength test was completed, and the sample was immediately placed in anhydrous ethanol, to terminate the hydration, for 7 days and then placed in a vacuum-drying oven at 60 °C to dry to a constant weight. The surface of the vacuum-dried sample was sprayed with gold to make it conductive and was then tested.

## 3. Results and Analysis

### 3.1. Shrinkage

**Figure 2:**
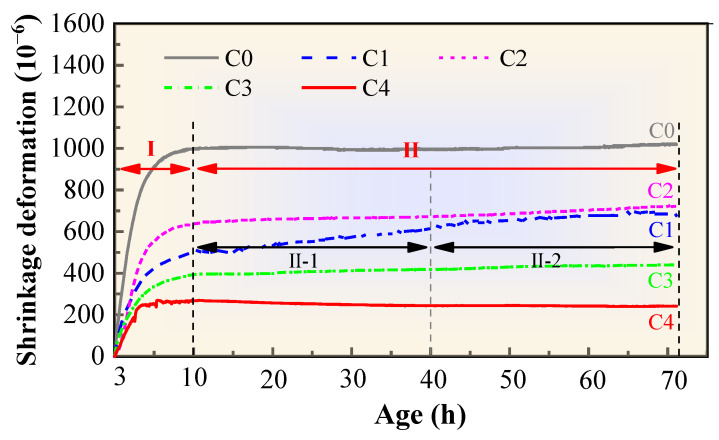
Shrinkage curves of the concretes.

The 3–72 h shrinkage curves of different mixtures are shown in Figure 2.

The early shrinkage curves of each of the concrete samples exhibit the same trend, which can be divided into the first and second stages. The first stage is mainly from the initial setting to the final setting stages of the concrete, and most of the cement will complete the hydration reaction in this stage. The water in the concrete is consumed in large quantities because of the rapid hydration of the cement at this stage, which results in the most obvious increase in the shrinkage rate. In the second stage, the development of autogenous shrinkage in the concrete gradually slows with decreasing cement hydration reaction speed. Moreover, its ability to resist shrinkage deformation is enhanced because of the hardening, which causes the shrinkage rate of the concrete in the second stage to gradually slow down. This is also the main reason the increase in the shrinkage rate at the second stage obviously slows down.

The shrinkage values of the C0, C1, C2, C3, and C4 concretes after 72 h are 1025 × 10^−6^, 680 × 10^−6^, 720 × 10^−6^, 440 × 10^−6^, and 241 × 10^−6^, respectively. The shrinkage values of the C1, C2, C3, and C4 concretes decreased by 33.66%, 29.76%, 57.07%, and 76.49% compared to the C0 concrete. The data showed that FS, SRA, PVA, and SAP can effectively inhibit the early shrinkage strain. The most effective shrinkage reduction method is to add SAP, followed by PVA, and the addition of an appropriate amount of FS and SRA has a similar shrinkage reduction inhibition effect on the MS concrete. The reasons are as follows.

First, the SAP has the greatest effect on reducing the shrinkage, mainly because the SAP has special water absorption and release characteristics. The water release effect of the SAP reduces the capillary pressure of the cement paste during the process of concrete hardening, which is helpful for improving the plastic shrinkage cracking caused by water loss. Wang et al. [37] also showed that the incorporation of SAP can significantly delay the decrease in the internal humidity, thereby improving the autogenous shrinkage of the concrete. In addition, Figure 2 shows that the shrinkage curve of the concrete with pre-absorbent SAP clearly fluctuates, which is different from what is observed for the other concrete samples. This is because the water pre-absorbed by the SAP supplements the capillary pores of the concrete in the initial setting–final setting stage so that a part of the shrinkage is restored, and the curve fluctuates. This conclusion is consistent with that of Yu et al. [21].

Second, the inhibitory effect of PVA fibers on concrete shrinkage is better than those of FS and SRA, mainly because the fibers can improve the pore structure of the concrete at different scales and levels in three-dimensional space, refine the size of capillary pores, and improve the uniformity of the concrete, thus effectively preventing the dissipation of water and reducing the capillary shrinkage stress. Furthermore, the three dimensions of the PVA fibers in concrete can increase the tensile stress inside the concrete, and some capillary shrinkage stress can be reduced or eliminated by the traction force applied between the fibers and cement matrix, which is another main reason fibers can effectively reduce the shrinkage rate.

Third, the FS and SRA had similar inhibitory effects on the shrinkage of the MS concrete with the high-volume SP, but the FS was slightly better than the SRA. The reason is that the equal mass substitution of the FS for the cement reduces the proportion of the cement per unit of volume. Although both F and S have certain hydration activities and pozzolanic activities, they are significantly lower than those of the cement, which is the main reason for the obvious decrease in the shrinkage rate of the C1 concrete in the first stage. In addition, the slag powder contains a certain number of microcrystals and a large amount of vitreous structure, and the fly ash contains active SiO_2_ (vitreous SiO_2_), active Al_2_O_3_ (vitreous Al_2_O_3_), and free CaO. Those components undergo secondary hydration reactions under certain alkaline conditions (caused by the cement hydration reaction in stage I), which is the reason C1 concrete can still maintain a high rate of shrinkage increase in the early stage of the second stage (II-1). The number of active FS particles continues to decrease with further hydration (II-2); the speed of the secondary hydration reaction slows down, and the increased rate of concrete shrinkage also tends to increase gradually. Significantly, the FS composite admixture has better powder gradation and microaggregate-filling effects, which can improve the particle gradation (especially the cementitious material) and the pore structure and improve the compactness of the concrete and reduce its early-age capillary pressure. This is another important reason the FS composite admixture can reduce the shrinkage rate of the concrete at an early age.

Finally, the action mechanism of the SRA mainly consists of the following two aspects: First, the reduction in the shrinkage stress generated when the capillary pores lose water is reduced by decreasing the pore water surface tension of the concrete; second, the viscosity of the pore water is increased by the SRA to enhance the adsorption of water molecules in the gel and further reduce the final shrinkage value of the concrete [21]. However, the water consumption of the concrete mixture used in this test is low, so the amount of internal pore water is relatively low during the hydration process, and the effect of the SRA is limited. In addition, the dose of the SRA used in this study may not be the optimal dose for this mixture, which may also explain why the effect of the SRA was limited.

### 3.2. Cracking

**Figure 3:**
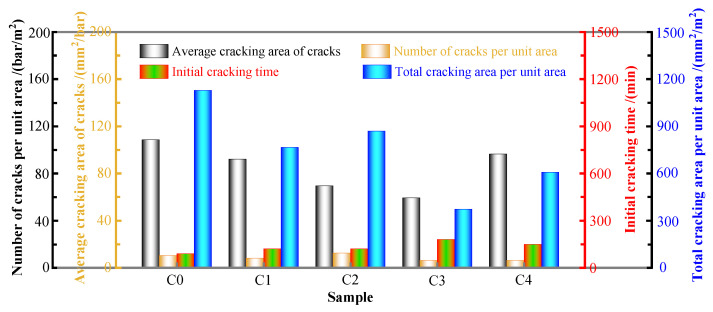
Cracking test results for concretes.

Table 4 and Figure 3 show the early-age cracking test results for the concrete.

Compared to those of the C0 concrete, the four abovementioned measures can delay the *t* of the mixtures. The additions of FS and SRA delay the initial time of the cracking to 120 min; the incorporation of PVA fibers delays the t of the concrete to 180 min, and the incorporation of SAP delays *t* to 150 min. In addition, the *a*, *b*, and *c* values of the concrete are significantly reduced by the four abovementioned measures. The total cracking areas of the C1, C2, C3, and C4 concretes are reduced by 32.22%, 23.02%, 66.91%, and 46.16%, respectively, compared to the C0 concrete. In general, the four abovementioned measures can effectively reduce the cracking risk. The anti-cracking effect of the PVA fibers is the best, followed by that of SAP, and those of FS and SRA, which have similar cracking inhibition effects on MS concrete. The reason is that the four abovementioned measures can effectively decrease the early-age shrinkage of the concrete, and the risk for cracking decreases with decreasing shrinkage.

It is worth noting that although the SAP has a better shrinkage reduction effect than the PVA fibers, the latter have a better cracking inhibition effect on the concrete than does the SAP. The reason is that the SAP can help to improve the plastic shrinkage cracking caused by water loss through water absorption and release characteristics and can form a microreservoir inside the concrete [38]. This microreservoir is supplemented to a certain extent after the evaporation of water on the surface of the specimen, which reduces and delays the expansion of cracks to a certain extent. It can be determined that the SAP mainly achieves the effect for inhibiting concrete cracking by reducing the cracking risk of the concrete. However, white PVA fibers can be observed at the cracks in the concrete mixed with sample C3 during the test. These fibers are within the crack development path, and the increase in the crack size is limited, which inhibits their development into macroscopic cracks, thus improving the crack resistance ability of the concrete. Therefore, PVA fibers can not only reduce the cracking risk but also improve the cracking resistance ability of the concrete by limiting the expansion of macrocracks, which is the reason the PVA fibers have a better cracking inhibition effect.

The cracking inhibition effects of the FS and SRA on the MS concrete are similar, and the FS and SRA can effectively reduce the cracking risk and early-age shrinkage. The incorporation of the SRA obviously causes more microcracks to form in the concrete, but the crack width is significantly reduced. This is mainly because the SRA reduces the surface tension of the concrete. A decrease in surface tension indicates a decrease in the negative capillary pressure, thereby reducing the expansion of cracks. Although the SRA concrete has more cracks, its average cracking area and total cracking area are significantly lower than those of the C0 concrete, indicating that the SRA still has a good cracking inhibition effect on the concrete.

### 3.3. Compressive Strength

**Figure 4:**
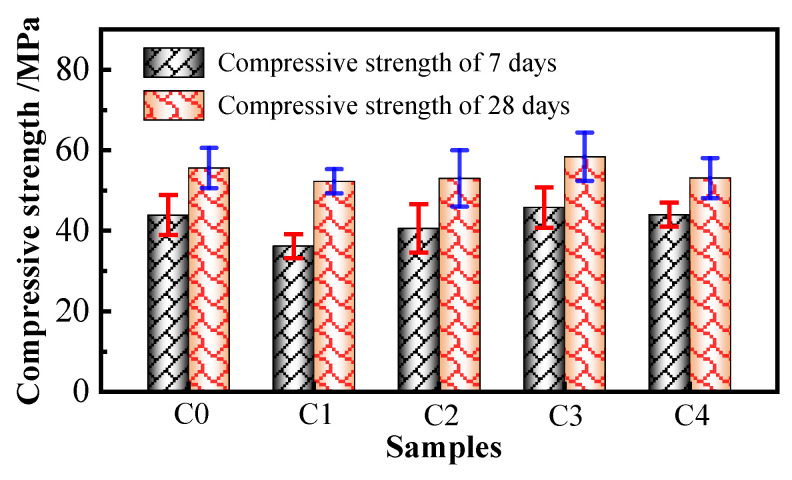
Compressive strength test results for concretes.

The *f*_cu_ values of the different concrete samples after 7 and 28 days are shown in Figure 4.

Compared to those of the C0 concrete, the changes in the *f*_cu_ values at 7 days for the C1, C2, C3, and C4 concretes were −17.54%, −6.83%, 4.33%, and 0.23%. The changes in the *f*_cu_ values at 28 days were −6.47%, −4.68%, 5.04%, and −4.50%. It can be seen that the FS has a significant adverse effect on the 7-day strength of the concrete, but its effect at 28 days is not obvious. This is mainly because the equal mass substitution of the FS for the cement reduces the proportion of the cement per unit of volume, slows the overall cement hydration reaction in the early stage, and significantly reduces the *f*_cu_ value of the concrete at an early age. However, microcrystals and a large number of vitreous structures in the FS began to participate in the secondary cement hydration reaction with increasing age, which further improved the compactness of the concrete, and the *f*_cu_ values of the 28-day concrete improved. The influences of the SRA and SPA on the *f*_cu_ values of the concrete are slightly reduced, and these effects are not significant. This finding is consistent with the experimental conclusions of Yu et al. [21] and Zhong et al. [39]. The fibers restrict the lateral deformation of the concrete to a certain extent when the concrete is compressed; thus, the fibers improve the 7- and 28-day strengths of the concrete. However, the effect of a small number of fibers on improving the *f*_cu_ value of the concrete is not obvious. In general, the addition of the FS leads to a significant decrease in the early-age *f*_cu_ value of the concrete, but its effect on the later *f*_cu_ value is not significant. In addition, although the influences of the SRA and SPA on the concrete strength are slightly reduced, that of the PVA fibers has a slight enhancing effect on the concrete strength, and the total influence of these four factors on the *f*_cu_ value is less than 6%. Therefore, the influences of the above methods for inhibiting shrinkage and cracking on the *f*_cu_ value of the concrete are not particularly important in engineering applications.

### 3.4. Flexural Strength

**Figure 5:**
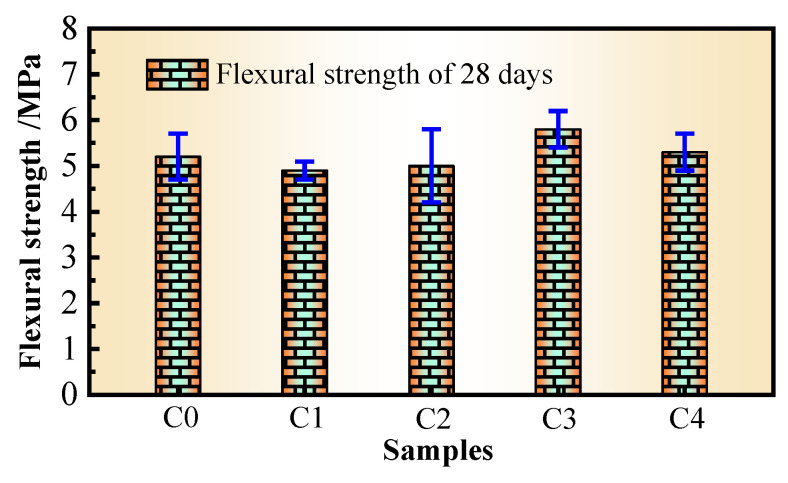
Flexural strengths of the concretes.

The test results of the *f*_f_ values of the concrete at 28 days are shown in Figure 5.

Similarly, compared with those of C0, the changes in the *f*_f_ values of the C1, C2, C3, and C4 concretes are −5.77%, −3.85%, 11.54%, and −1.92%, respectively. The data show that the FS, SRA, and SPA have little effect on the *f*_f_ values, and the reason and mechanism are the same as those for the above *f*_cu_ values. Although PVA fibers have certain improvement effects on the compressive strengths and *f*_f_ values, the improvement in *f*_f_ values by PVA fibers is much greater than that in the *f*_cu_ values. The reason is that because of the good bonding force between the PVA fibers and hardened cement paste, the fibers restrict the transverse deformation of the concrete to a certain extent, when the concrete is compressed to improve its *f*_cu_ value. However, the effect of these constraints is limited, and the improvement in the concrete’s *f*_cu_ values is not obvious. However, once the cement matrix is slightly cracked when the concrete is bent, the bond between the fibers and the matrix will limit the rapid development of cracks until the fibers are pulled out or broken, which greatly improves the ductility and toughness of the matrix. Therefore, the fibers can bear most of the tensile stress when the concrete is bent, thereby obviously improving the *f*_f_ value.

### 3.5. Electric Flux

**Figure 6:**
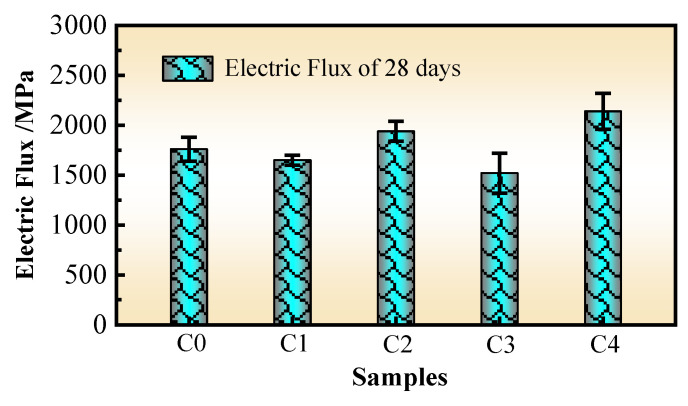
Electric fluxes of concretes.

The electric fluxes of the concretes at 28 days are shown in Figure 6.

The higher the electric flux value of the concrete, the more chloride ions pass through it per unit of time, and the worse the penetration resistance of the concrete is. In contrast, this result shows that the penetration resistance is better. Figure 6 shows that compared with those of the C0 concrete, the 28-day electric flux changes in the C1, C2, C3, and C4 concretes are −6.25%, 10.23%, −13.64%, and 21.59%, respectively. The electric flux values of the five samples of concrete measured in the test are all greater than 1500 C. The reason is that the higher SP content has a dilution effect, microcrack effect, and free effect on the impermeability of the concrete, which leads to relatively poor penetration resistance performance [1].

The test data show that the FS has a certain optimization effect on the penetration of the concrete. The reason is that although the equal mass replacement of the cement by the FS slows down the early cement hydration reaction and significantly reduces the early-age strength of the concrete, microcrystals and a large number of vitreous structures in the fly ash and slag powder begin to participate in the secondary cement hydration reaction in the later stage, which improves the compactness of the concrete and restores its strength after 28 days of curing. In addition, the FS has good powder gradation and microaggregate-filling effects, which can improve the particle gradation of powder materials (including the SP and cement) and the pore structure of the concrete and effectively decrease the probability for connecting micropores in the concrete. This is the main reason the FS can improve the penetration resistance.

In addition, the test showed that PVA fibers have a certain optimization effect on the penetration resistance of the concrete. The main reasons may be as follows: First, PVA fibers are randomly distributed in three dimensions in the concrete and have a good bonding force with the cement matrix, which can effectively decrease the number and connectivity probability of micropores caused by dilution, microcracks, and free effects from the SP in the MS concrete with the high-volume SP. Second, PVA fibers can better inhibit the development of microcracks in the early stage of the concrete specimen formation, reduce the generation of internal and external connected pores, and enhance the internal binding force of the concrete. Therefore, PVA fibers have a certain improving effect on the impermeability of the concrete.

Significantly, the test results show that both the SRA and SPA lead to the deterioration of the penetration resistance of the concrete. The main working mechanism of the SRA is to increase the viscosity of the pore water, thereby enhancing the adsorption of water molecules in the gel. Although this approach can effectively reduce the shrinkage rate and cracking area of the concrete, it is likely to lead to an increase in small cracks near the coarse aggregate (because the coarse aggregate has a certain constraint during the shrinkage process. See Section 3.2 above). Similarly, the water absorption–release characteristics of the SAP and tiny reservoir effects can reduce the shrinkage rate and cracking area of the concrete, but water release inevitably leads to an increase in micropores. It should be noted that although the increase in the number of micropores and cracks caused by the SRA or SAP may not have a significant effect on the strength, chloride ions, with a radius of 0.181 nm, are sufficient to migrate in these micropores and cracks. There is a large number of microcracks and free SP inside the MS concrete with the high-volume SP, and an increase in micropores will inevitably lead to an increase in the probability of internal connected holes.

### 3.6. Nuclear Magnetic Resonance

The results of the NMR tests are represented by *T*_2_ spectrum curves, and the area under the *T*_2_ peak is linearly related to the pore water content [40,41,42]. The transverse relaxation time (*t*_2_) of the pore water in the concrete can be expressed as

1/*t*_2_ ≈ *ρ*_2_ *S*/*V*

where *ρ*_2_ is the surface relaxation strength, and *S* and *V* are the pore surface area and pore volume, respectively.

**Figure 7:**
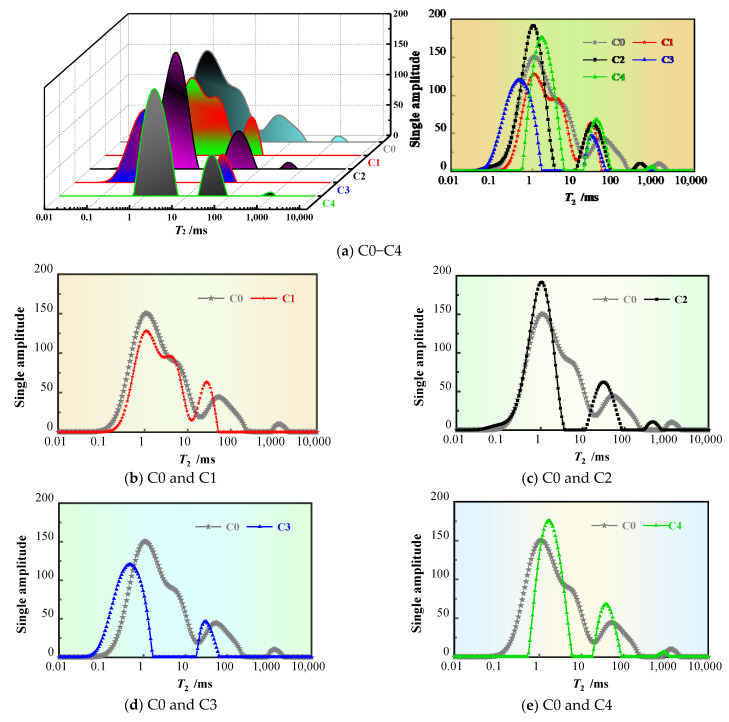
*T*_2_ spectral distributions of concretes.

Figure 7 shows the *T*_2_ spectral distribution curves of the different concretes. The abscissa of the curve represents the relaxation time, which reflects the pore size.

The shorter the relaxation time, the smaller the pore size is. It can be seen from Figure 7a that the spectra for C0, C2, and C4 have three peaks, while those for C1 and C3 have only two peaks. These findings show that the FS and PVA fibers have obvious optimization effects on large pores and can reduce the number of large pores in concrete. In addition, the first and second signal peaks in the spectra of the concrete mixed with the FS, SRA, PVA fibers, and SAP are obviously shifted to the left compared to those in the spectrum of the C0 concrete (and the corresponding relaxation times at these peaks are shorter), and the peak area is significantly reduced, indicating that the coarse pores in the concrete are transformed into tiny pores and that the pore structure is optimized, which has an inhibitory effect on the development of cracks and pores in the concrete.

In Figure 7b–e, it can be seen that the incorporation of the FS and PVA fibers can effectively reduce the *T*_2_ peak and total peak areas in the spectra of the concrete so that the *T*_2_ spectrum curve of the concrete shifts to the right and decreases obviously. These findings show that the FS and PVA effectively optimize the pore structure of the concrete, which can obviously improve its compactness and reduce its porosity. This is also the main reason it can improve the impermeability of the concrete. The optimization effect of the PVA fibers on the pore structure of the concrete is more significant than that of the FS according to the comparison of the *T*_2_ spectral curves of the C1 and C3 concretes, which explains why the C3 concrete has better penetration resistance.

It is worth noting that compared to that in the C0 spectrum, although the total peak area in the spectra of the concretes mixed with the SRA and SAP decreased slightly, the first and second peak strengths increased slightly. In addition, the first *T*_2_ peak in the spectrum of the SAP concrete sample exhibited a small rightward shift, which may be because of an increase in the micropores in the concrete caused by the SAP water release. Although the addition of the SRA or SAP reduced the internal porosity to a certain extent, it indeed led to a slight increase in the proportion of micropores in the concrete. An increase in micropores and cracks may not have a significant effect on the strength of ordinary concrete, but it has a more obvious adverse effect on the penetration resistance of the high-volume-SP concrete. This is because there is a large number of microcracks and free SP inside the MS concrete with the high-volume SP caused by the diversification effect of the SP [1,10]. An increase in microcracks will inevitably lead to an increase in the probability of internal connected pores, which is the difference between high-SP-content concrete and ordinary concrete as well.

### 3.7. Scanning Electron Microscopy

**Figure 8:**
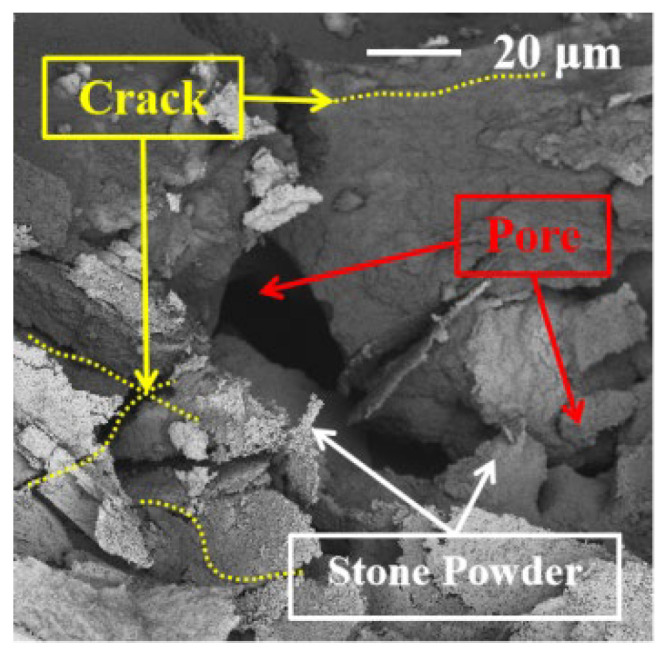
The SEM image of the C0 concrete.

**Figure 9:**
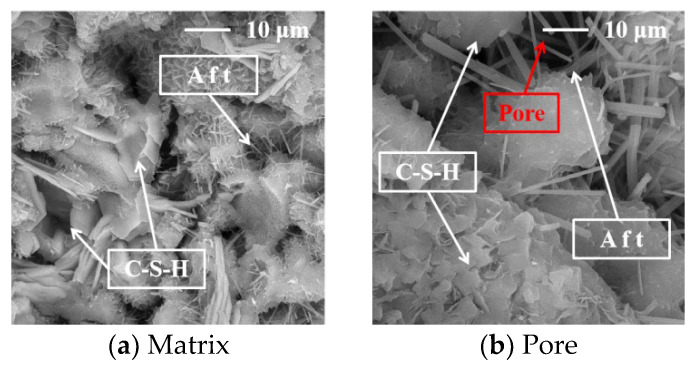
The SEM images of the concrete with the FS.

**Figure 10:**
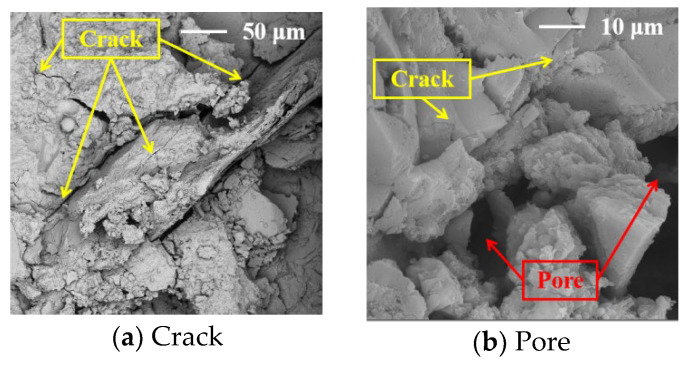
The SEM images of the concrete with the SRA.

**Figure 11:**
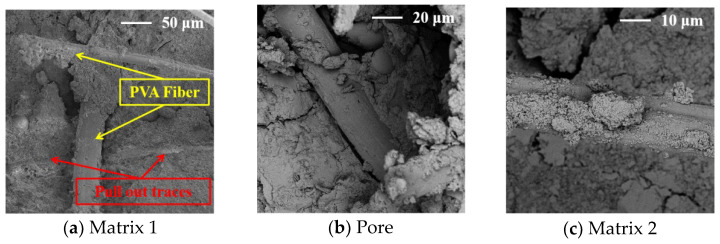
The SEM images of the concrete with PVA fibers.

**Figure 12:**
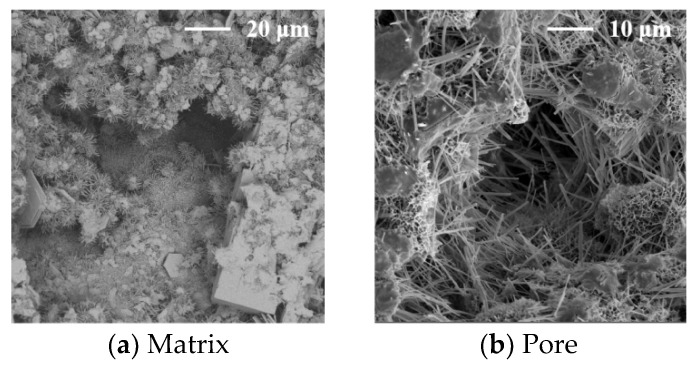
The SEM images of the concrete with the SAP.

The SEM images of the concrete at 28 days are shown in Figure 8, Figure 9, Figure 10, Figure 11 and Figure 12.

It can be clearly seen from Figure 8 that the internal pores in the C0 concrete are large, numerous, and contain many small cracks, which are caused by the high shrinkage of the C0 concrete. In addition, free SP is present in the C0 concrete, and the cement matrix is also relatively loose, mainly because a large amount of SP will lead to more interfacial transition zone defects and destroy the most closely packed structure of powder materials [1]. Those two points are the main reasons for the poor mechanical properties and impermeability of the MS concrete with the high-volume SP.

Figure 9a shows that the interior of the C1 concrete is relatively dense, and the pore structure and cracks are less dense and smaller. The composite admixture has a better powder gradation and a greater microaggregate-filling effect, which can improve the particle gradation, and the pore structure can effectively disperse the close accumulation of the SP to improve the compactness of the concrete [10,11,12,13]. In addition, fine FS particles not only cause nucleation and hydration reactions but also promote the formation of Aft and C-S-H in the later stage, which can effectively reduce the porosity, as shown in Figure 9b. This is the reason the FS improved the shrinkage and cracking penetration resistance of the concrete.

It can be seen from Figure 10 that although the pore size of the concrete mixed with the SRA is small, the number of pores and cracks is greater than those in the C0 concrete. The SRA is likely to lead to an increase in tiny cracks near the aggregate (see Section 3.2 above). In addition, studies [21] have shown that the SRA can reduce the internal stress of the concrete and affect the hydration reaction of the cement, resulting in more micropores in the matrix. This is the main reason it leads to a significant decrease in the impermeability of the high-volume-SP MS concrete.

Figure 11 shows that the interior of the C3 concrete is relatively dense, and the pore structure and cracks are less dense and smaller. The cement-based material is attached to the uneven surface of the fibers, and the fibers can provide a good carrier for the cement-based material (Figure 11a), increasing the density of the concrete. In addition, the tensile trace left by the fibers on the cement matrix indicates that the fibers can enhance the bonding force between the particles inside the concrete (Figure 11a,b) and fill the pores inside the concrete (Figure 11c), which has a good inhibitory effect on the loss of the water and reduces the generation of microcracks inside the concrete. This is also the main reason it can reduce the shrinkage of the concrete and reduce the width and area of concrete cracks.

It can be seen from Figure 12 that a large amount of ettringite is formed inside the C4 concrete. Few cracks are found in the cement matrix, and there may be large holes in the local area. The water–cement ratio around the SAP particles is high after the water pre-absorbed by the SAP is released, which promotes the formation of ettringite. The volume decreases after the SAP is dehydrated, which provides good space for the formation of ettringite so that the degree of cement hydration is improved. The expansion and filling of ettringite improve the early shrinkage, crack resistance, and early-age strength (7 days) of the concrete. However, the water pre-absorbed by the SAP is completely released with increasing concrete age, but the formation of ettringite obviously slows, or even stops, and the volume of ettringite generated is insufficient to reduce the volume of the SAP water loss, leading to increases in the pore volume and number in the concrete. This is the reason for the slight decrease in the 28-day strength and durability of the C4 concrete.

## 4. Conclusions

The MS concrete with the high-volume SP was taken as the research object in the tests, and the effects of the FS, SRA, PVA fibers, and SAP on the early shrinkage, cracking, strength, and penetration resistance of the concretes were reported. Furthermore, the microstructures and pore structures of the different concretes were tested using NMR and SEM, and the effect mechanisms were revealed.

1. The incorporation of the FS, SRA, PVA fibers, and SAP can inhibit the shrinkage and cracking of the concrete at an early age. Among them, the pre-absorbent SAP has the greatest effect on reducing the early shrinkage, and the shrinkage strain can be reduced by 76.49%. The effect of PVA fibers on inhibiting early cracking was the most obvious, and the total cracking area was reduced by 66.91%;
2. The incorporation of the FS can improve the particle gradation and the pore structure and improve its compactness. The shrinkage rate and cracking area of the FS concrete decreased by 33.66% and 32.22%, but the changes in the strength and impermeability did not seem to be obvious;
3. The SRA can reduce the early-age shrinkage and cracking risk, but it will lead to significant decreases in the later strength and impermeability of the high-volume-SP concrete. The shrinkage rate and cracking area of the SRA concrete decreased by 29.76% and 23.02%, and the *f*_cu_ and *f*_f_ values and electric flux increased by −6.47%, 3.85%, and 10.25%;
4. The incorporation of PVA fibers will not only improve the early-age crack resistance and reduce the shrinkage of the concrete but also lead to improvements in the later strength and impermeability of the concrete. The shrinkage rate and cracking area of the PVA concrete decreased by 57.07% and 66.91%, and the *f*_cu_ and *f*_f_ values and electric flux increased by 5.04%, 11.59%, and −10.25%;
5. The shrinkage rate and cracking area of the SAP concrete decreased by 76.49% and 46.16%, and the *f*_cu_ and *f*_f_ values and electric flux increased by −4.5%, 1.92%, and 21.59%. The water absorption and release characteristics of the SAP can promote the rapid formation of Aft at an early age, and the expansion and filling effect of the Aft can improve the early shrinkage, early cracking, and early strength of the concrete. However, the water pre-absorbed by the SAP is completely released with increasing age, and the volume of the Aft that is generated is not enough to compensate for the decrease in the volume associated with the SAP water loss, which leads to significant decreases in the strength and impermeability of the high-volume-SP concrete.

## Figures and Tables

**Table 1:** The properties of the cement.

Soundness	Setting Time/min		Specific Surface Area /(m^2^/kg)	Flexural Strength/MPa		Compressive Strength/MPa	
	Initial	Final		3 Days	28 Days	3 Days	28 Days
Qualified	260	330	355	4.9	7.2	23.1	48.1

**Table 2:** The properties of the MS.

Fineness Modulus	Apparent Density /(kg/m^3^)	Bulk Density /(kg/m^3^)	SP Content/%	Loose Density /(kg/m^3^)	Voidage /%	Crushing Index /%
2.8	2630	1740	10	1630	38	10

**Table 3:** Mixture proportions of concretes (kg/m^3^).

Sample	Cement	MS	Crushed Stone	Water	Superplasticizer	FA	GGBFS	SRA	PVA	SAP
C0	500	679	1061	160	6.0	—	—	—	—	—
C1	350	679	1061	160	6.5	100	50	—	—	—
C2	500	679	1061	160	6.0	—	—	7.5	—	—
C3	500	679	1061	160	6.5	—	—	—	2.6	—
C4	500	679	1061	160	7.5	—	—	—	—	1.0

**Table 4:** Cracking test results for concrete.

Sample	*t* /h	*a* /(mm^2^/bar)	*b* /(bar/m^2^)	*c* /(mm^2^/m^2^)	*n* /%	Crack Model
C0	90	108.60	10.4	1129.44	—	Mostly along a knife edge and a small amount of clutter
C1	120	92.23	8.3	765.51	32.22	More along a knife edge
C2	120	69.56	12.5	869.44	23.02	A small amount along a knife edge and more surface microcracks
C3	180	59.33	6.3	373.78	66.91	A small amount along a knife edge and basically no clutter cracks
C4	150	96.52	6.3	608.08	46.16	More along a knife edge

Variables *t*, *a*, *b*, and *c* represent the initial cracking time, average cracking area, number of cracks per unit of area, and total cracking area per unit of area of concrete sample, respectively; *n* representing the crack reduction factor.

## Data Availability

Data are contained within the article.
